# Experimental translocations to low predation lead to non-parallel increases in relative brain size

**DOI:** 10.1098/rsbl.2019.0654

**Published:** 2020-01-22

**Authors:** David J. Mitchell, Regina Vega-Trejo, Alexander Kotrschal

**Affiliations:** 1 Department of Zoology/Ethology, Stockholm University, Svante Arrheniusväg 18B, 10691 Stockholm, Sweden; 2 Department of Animal Sciences, Behavioural Ecology group, Wageningen University, 6708 Wageningen, The Netherlands

**Keywords:** telencephalon, cognition, convergent evolution, predator–prey interactions

## Abstract

Predation is a near ubiquitous factor of nature and a powerful selective force on prey. Moreover, it has recently emerged as an important driver in the evolution of brain anatomy, though population comparisons show ambiguous results with considerable unexplained variation. Here, we test the reproducibility of reduced predation on evolutionary trajectories of brain evolution. We make use of an introduction experiment, whereby guppies (*Poecilia reticulata*) from a single high predation stream were introduced to four low predation streams. After 8–9 years of natural selection in the wild and two generations of common garden conditions in the laboratory, we quantified brain anatomy. Relative brain region sizes did not differ between populations. However, we found a general increase and striking variation in relative brain size of introduced populations, which varied from no change to a 12.5% increase in relative brain weight, relative to the ancestral high predation population. We interpret this as evidence for non-parallel evolution, which implies a weak or inconsistent association of relative brain size with fitness in low predation sites. The evolution of brain anatomy appears sensitive to unknown environmental factors, or contingent on either chance events or historical legacies of environmental change.

## 1. Introduction

Predation is a powerful selective pressure, leading to directional selection on a diverse range of traits, including life history [1,2], morphology [3] and behaviour [4,5]. Such differences can evolve remarkably fast, often within few generations, in both the laboratory [2,6] and in the wild [1,7]. Recently, the effect of predation on brain evolution has begun to be unveiled [8–10]. Larger brains are often associated with better executive function than smaller brains [11,12], which has been experimentally shown in guppies selected for relative brain size [13–17]. These selection lines highlighted the potentially strong selective pressure of predation on brain size, as large-brained guppies exposed to predators survived better than small-brained guppies [18], potentially owing to behavioural advantages [17]. However, large brains are also energetically costly [13,19–21] and this high energy demand may increase the required foraging rate and therefore the exposure to predators [22].

A series of recent ecological comparisons have begun to elucidate the effect of predation on brain evolution. These have focused on the Trinidadian streams, where pronounced geographical variation in predation risk occurs in close proximity [23]. However, the results and conclusions contrast greatly, as positive [9], negative [10] and no associations [8,24,25] between brain size and predation regimes were reported. These studies made use of the natural variation in predation regimes to infer evolution *post hoc*. Alternatively, through experimental manipulations of the environment, such as translocating populations, we can study the process of evolution, while maintaining natural trade-offs (e.g. between predator avoidance and foraging activity to meet energetic requirements) [26]. With this approach, we can test whether the evolution of brain morphology is repeatable. This is important as the presence or absence of parallel (or convergent) evolution implies adaptation of the trait's response [27]. Alternatively, evolution may be contingent on historical factors or chance events. For instance, replicate populations may each increase their fitness to the new conditions, while a specific trait that does not strongly affect fitness may diverge among populations [28]. Translocation experiments are hence a powerful tool to decipher whether observed brain anatomy differences between populations are a result of adaptation, or contingent on chance events and historical factors.

Here, we make use of a long-term experimental evolution field manipulation, where guppies from a single high predation site were translocated to four low predation sites. Previous studies have found rapid evolution in morphology, colouration and life history [29–33]. We expected a reduction in brain size of translocated fish owing to the reduced benefits conferred by larger brains when under predation [8,17] and greater energetic constraints of high population densities in low predation sites.

## 2. Material and methods

### (a) Source populations

Fish were derived from a translocation experiment, carried out in 2008 and 2009, where guppies were translocated from the high predation locality in the Guanapo river, characterized by the presence of Pike cichlids (C*renicichla frenata*), to low predation sites where Pike cichlids are absent. In 2008 juvenile guppies were caught in the Guanapo, reared and mated in the laboratory, and 38 fish of each sex were introduced to two streams (Upper and Lower Lalaja). In 2009, this experiment was replicated, where guppies were again derived from the Guanapo and introduced to the Taylor (*N* = 52 males and 52 females) and Caigul rivers (*N* = 64 males and 64 females). Females and the males they were mated with were not sent to the same stream, so effective population sizes were larger than these numbers suggest owing to sperm storage by females. These translocations of guppies thus come from the same high predation population which largely standardized the gene pool of founders. For concurrently running experiments, the canopy of the Upper Lalaja and Taylor were regularly thinned to let through more light and resemble the light environment of the high predation populations downstream. More information about these translocations can be found in Handelsman *et al*. [30] and descriptions of the ecological processes following the introduction are reviewed in [32,33].

### (b) Sampling

A sample of 40–50 juveniles from the four introduction sites and the origin population were returned to the laboratory at the University of California Riverside in 2016, 8–9 years after translocations. Quantitative genetic analyses performed on the Lower Lalaja population estimated approximately 1.7 generations per year (≈15 generations) [34]. Fish were kept for two generations in tanks of 5–6 individuals in an 8 L tank under common garden conditions to ameliorate potential non-genetic developmental or parental effects. Previous studies have shown plastic responses of brain size to environmental heterogeneity [35] or directly to olfactory predator cues [9], which we tried to minimize by rearing fish in common garden conditions to focus on evolved change. During this time, fish were randomly outcrossed to create full-sibling fry. This excluded non-random mating, thereby reducing undesired selection in the first two generations. After this period, fish were placed in stock tanks and allowed to breed naturally for a little over 1 year, which may have dampened genetic differences among populations as they may evolve to laboratory conditions. Information on sampling and husbandry can be found in Reznick *et al*. [33]. The 114 fish used here, balanced for all populations and both sexes, were taken from these stock tanks and therefore at least two generations removed from the streams. Fish were euthanized with an overdose of tricaine methanesulfonate (MS-222; Sigma-Aldrich), fixed in 4% buffered formalin solution and shipped to Stockholm University for processing. Fifteen of those fish were discarded owing to fixation and dissection issues.

### (c) Dissections

Fish were placed under a dissection microscope (Leica MZFLIII), their standard length was measured with callipers (from the tip of the nose to the end of the caudal peduncle) and brains dissected out and stored in phosphate-buffered solution. We photographed the brains under the dissection microscope with an attached Leica DFC 490 camera from the ventral, dorsal and both lateral sides, and then weighed them to the nearest 0.01 mg (Mettler MT5 scale). The length, width and height of the olfactory bulbs, telencephalon, optic tectum, hypothalamus, cerebellum and dorsal medulla were measured with the ImageJ software [36], with protocols described in Kotrschal *et al*. [37]. These measures were used to estimate the volume of the brain region, as given by equation (2.1). All of these procedures were conducted by one observer (DJM) and image processing was done blind to the stream origin.

2.1 $$V=(L\times W\times H)\frac{\pi }{6}.$$

### (d) Statistics

To achieve normality and linearize the allometries between response variables and predictors, we log_e_-transformed body lengths (mm), brain weight (mg) and brain region volumes (mm^3^). Brain weight was fit with the predictors of population, sex, log_e_(length) and all two-way interactions, however, all interactions were uninformative to the model and were, therefore, removed (all *p* > 0.17; see electronic supplementary material). The brain region volumes were fit to a multivariate linear model, with the predictors of population, sex, log_e_(brain weight) and all two-way interactions. Again, all interactions were uninformative to the model and were discarded (all *p* > 0.6). Analyses of body size differences revealed fish from the Guanapo and Caigul were smaller than the other populations, though we caution against biological interpretations as fish are of an unknown age. Models were run in R statistics package [38], and group effects are compared with type-III ANOVAs.

## 3. Results

### (a) Brain weight

**Figure 1.**
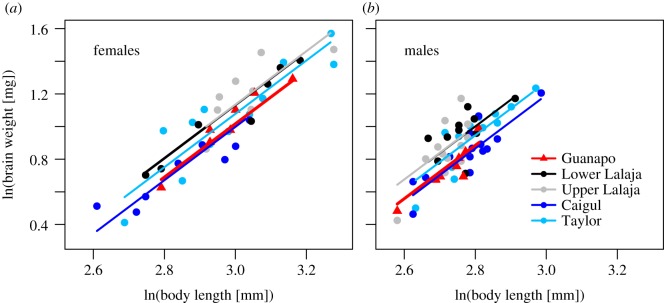
Population differences in relative brain size. Displayed is brain weight plotted against body length for female (*a*) and male (*b*) guppies. The reference high predation population Guanapo is highlighted in red. Upper Lalaja and the Taylor were the locations of the canopy thinning. Model predictions are plotted as the best fit line.

We found a strong allometry between brain weight and body length (estimate = 1.63, s.e. = 0.091, *F*_1,92_ = 326, *p* < 0.0001), and that males had 21.5% larger relative brain sizes than females (est. = 0.2, s.e. = 0.028, *F*_1,92_ = 47, *p* < 0.0001; figure 1). After accounting for these effects, there were substantial differences among populations in relative brain sizes (*F*_4,92_ = 6.93, *p* < 0.0001), with the Lower Lalaja (difference = 12.3%, est. = 0.12, s.e. = 0.037, *F*_1,92_ = 9.9, *p* = 0.002) and Upper Lalaja populations (diff. = 12.5%, est. = 0.12, s.e. = 0.035, *F*_1,92_ = 11.1, *p* = 0.001) showing an increase in brain size relative to the source Guanapo population, while the Caigul showed no change (diff. = −1.9%, est. = −0.019, s.e. = 0.035, *F*_1,92_ = 0.3, *p* = 0.58). The Taylor was intermediate, being insignificantly larger than the Guanapo (diff. = 6.3%, est. = 0.061, s.e. = 0.034, *F*_1,92_ = 3.2, *p* = 0.078), but also insignificantly different from the two Lalaja populations (*p* = 0.08 and 0.11). The thinning of the canopy in the Taylor and Upper Lalaja did not appear to affect brain size, as there was greater similarity within introduction years relative to between canopy type (see figure 1). Together, the results indicate that relative brain size increased from the source Guanapo population when introduced to three of the low predation sites.

### (b) Brain regions

The multivariate analysis of brain region volumes after accounting for the allometries associated with brain weight (*F*_6,87_ = 103, *p* < 0.0001) revealed a large effect of sex (*F*_6,87_ = 30.1, *p* < 0.0001), with males having a larger relative optic tectum, but smaller telencephalon, dorsal medulla and olfactory bulbs than females (see electronic supplementary material). There was no effect of population origin on brain region volumes (*F*_24,360_ = 0.6, *p* = 0.6), indicating population differences in brain sizes were equally expressed over the different brain regions.

## 4. Discussion

We found that guppy populations that were translocated from high to low predation sites evolved relatively larger brains, which seems to oppose previous results in this species [9]. Importantly, we also found considerable divergence in brain size among the introduction populations, which indicates non-parallel evolution. The variation in evolutionary trajectories of brain size was pronounced, with introduction sites ranging from no change (a 1.9% non-significant decrease) to a 12.5% increase in relative brain size, and cannot be explained by experimental canopy clearance, as the two cleared sites were quite different (figure 1).

This non-parallel evolution indicates that selective pressure on brain size, resulting from the difference in predation, was likely weak in the introduction sites [27,28]. This could mean either that other unobserved environmental factors may have contributed to the observed variation among populations or point to a role for evolutionary contingency leading to variation among introduction sites. However, it is important to note that the evidence for weak selection on brain anatomy in low predation populations does not preclude a strong effect of brain size on fitness in high predation populations. Rather, the results speak only to an effect of reduced predation pressure and the associated ecological changes.

This apparent weak selection speaks against previous adaptationist explanations for increased brain size in low predation environments. Low predation streams reach higher population densities and therefore have greater intraspecific competition. For instance, Walsh *et al*. [10] proposed that larger brains evolve in low predation populations of Rivulus killifish (*Rivulus hartii*) to assist in foraging and learning under high intraspecific competition. Introduction sites reached higher population sizes than the source site, with greater intraspecific competition and competition with Rivulus killifish [33]. It is possible that density and intraspecific competition did not have a direct effect on brain size in our system, or that changes in brain size owing to life-history evolution are not seen as rapidly. For our study, the ‘cognitive buffer’ hypothesis, which predicts larger brains to evolve to facilitate rapid responses to environmental change [39], may help explain our results. Fish were first introduced to novel conditions, which then underwent a series of ecological changes as guppy populations and densities shaped the ecological conditions [32]. However, these cognition-linked hypotheses would predict a more direct effect of brain size and hence promote more parallel evolution among populations than was observed.

A more parsimonious explanation of the increase may be a relaxation of selection on the constraints to the upper and lower limit of brain size in the initial period after the introductions. Large brains are energetically costly [13,19–21] and the initial period after the introduction was a period of relaxed energetic pressures, as guppy populations were under carrying capacity with abundant food availability [32]. Once populations were subject to density regulations, selection pressures switched from fast to slow growth [33], with fry born at a larger size [40], as resource availability plays a major role in shaping the evolution of growth rates [41]. As brain size is measured as being relative to body size, it is possible that our results reflect these life-history changes [42], with a lag in brain anatomy evolution. Such an explanation would be consistent with the observations in mice of a greater positive correlation of prenatal and early postnatal growth with brain size [43].

While we do not know how brain anatomy responded to density change, it is notable that the sites which showed the largest increases in brain size were the sites where carrying capacity was reached most slowly; the Lalaja introductions reached peak density more slowly (≈ 30 months) than the Taylor and Caigul (≈ 18 months) [33]. The observed population variation may, therefore, reflect this prolonged period of population expansion, before selection pressures changed under density-dependent selection. While this explanation is highly speculative, the data here, and published elsewhere [33,44], point to an important role for environmental factors other than predation (e.g. density and intraspecific competition), both contemporary and historical. These factors may aid in explaining the discrepancies among studies on brain size evolution in response to predation [8–10,24].

The evolution of brain anatomy is likely mediated by selection on behaviour and multiple trajectories may, therefore, create similar outcomes. For instance, the telencephalon is associated with ‘higher order’ executive function such as learning and memory [45,46]; therefore, selection favouring increased cognitive faculties could lead to larger relative telencephalon size, without an increase in overall brain size. However, we found no indication that this was the case in our data, as we found no evidence for brain region volume evolution between populations. Similarly, an increase in neuronal densities could increase the cognitive abilities of animals [47], while not affecting total brain size. Guppies show considerable among-individual variation in neuronal densities, which appears to evolve independently from brain size [48]. This may, therefore, potentiate rapid evolution in neuronal densities, yielding an alternative trajectory with a similar effect on behaviour and learning.

The apparent lack of evolution in brain region sizes may be considered additional support for a nonadaptive explanation to the changes in relative brain size. In addition to the hypothesized selection on telencephalon size discussed above, we may also expect selection on other brain regions. For instance, the translocation of fish to low predation streams increases the importance of sexual selection in low predation environments [6,29] and may relax selection of motor activity owing to the decreased importance of predator escape. As the cerebellum is important to motor action function and is integrated with sensory information in the optic tectum [46] we may have expected a change in its size, which we did not observe.

Here, we found a general increase of brain size of fish released from predation pressure, contrary to previous results of population comparisons [9]. Results from laboratory conditions indicate an advantage of large brains in predator populations [17,18], and it is possible that translocation from low to high predation may promote more convergent evolution. To investigate such effects of increased predation on brain evolution, either translocations of predators to previously predator-free populations (e.g. [49]) or replicated experimental evolution in the laboratory (e.g. [6]) would be required.

## Supplementary Material

Analysis Code

## Supplementary Material

Analysis Code

## Data accessibility

All data are available in the electronic supplementary material.
