# Supplementary material for: Experimental translocations to low predation lead to non-parallel increases in relative brain size
Source: Biol Lett. 2020 Jan 22;16(1):20190654. doi: 10.1098/rsbl.2019.0654 (PMC7013489; doi:10.1098/rsbl.2019.0654)
Supplement: Analysis Code [file rsbl20190654supp1.pdf]

Brain evolution in the wild: Analysis output

David J. Mitchell

02/09/2019

Here I will show the model outputs of the univariate analysis of relative brain size and the multivariate analysis relative brain region sizes. First I ln-transformed and centred body length, and made the high predation source populationt he reference group.

```
ds$lgth <- log(ds$Length_mm) - mean(log(ds$Length_mm))
ds$Population <- relevel(ds$Population, ref = 'Guanapo')
ds$y <- cbind(log(ds$TelVol), log(ds$OptTectVol), log(ds$HypVol),
              log(ds$CerVol), log(ds$DorsMedVol),log(ds$OlfBulbVol))

mass <- lm(log(BrainMass_mg) ~ Population +Sex+ lgth,
           data = ds)
summary(mass)
```

```
##
## Call:
## lm(formula = log(BrainMass_mg) ~ Population + Sex + lgth, data = ds)
##
## Residuals:
##       Min        1Q    Median        3Q       Max
## -0.242824 -0.055504 -0.002789  0.065785  0.235103
##
## Coefficients:
##                       Estimate Std. Error t value Pr(>|t|)
## (Intercept)            0.76839    0.03038  25.289  < 2e-16 ***
## PopulationCaigul      -0.01907    0.03461  -0.551  0.58302
## PopulationLowerLalaja  0.11556    0.03671   3.148  0.00222 **
## PopulationTaylor       0.06096    0.03421   1.782  0.07806 .
## PopulationUpperLalaja  0.11773    0.03532   3.333  0.00124 **
## SexMale                0.19515    0.02842   6.868 7.53e-10 ***
## lgth                   1.63526    0.09052  18.066  < 2e-16 ***
## ---
## Signif. codes:  0 '***' 0.001 '**' 0.01 '*' 0.05 '.' 0.1 ' ' 1
##
## Residual standard error: 0.1052 on 92 degrees of freedom
## Multiple R-squared:  0.8258, Adjusted R-squared:  0.8145
## F-statistic: 72.69 on 6 and 92 DF,  p-value: < 2.2e-16
```

And here is the multivariate analysis of brain region sizes. Anova of full model is shown with type III sums of squares (using `car` package), then the effect on each brain region.

```
ds$y <- cbind(log(ds$TelVol), log(ds$OptTectVol), log(ds$HypVol),
              log(ds$CerVol), log(ds$DorsMedVol),log(ds$OlfBulbVol))
regions <- lm(y ~ Population +Sex+ log(BrainMass_mg),
          data = ds)
Anova(regions, type = 'III')
```

```
##
## Type III MANOVA Tests: Pillai test statistic
##                     Df test stat approx F num Df den Df Pr(>F)
```

```
## (Intercept)        1   0.96581   409.61      6     87 <2e-16 ***
## Population         4   0.22732     0.90     24    360 0.5972
## Sex                1   0.67463    30.06      6     87 <2e-16 ***
## log(BrainMass_mg)  1   0.87654   102.95      6     87 <2e-16 ***
## ---
## Signif. codes:  0 '***' 0.001 '**' 0.01 '*' 0.05 '.' 0.1 ' ' 1
```

```
summary(regions)
```

```
## Response Y1 :
##
## Call:
## lm(formula = Y1 ~ Population + Sex + log(BrainMass_mg), data = ds)
##
## Residuals:
##      Min       1Q   Median       3Q      Max
## -0.31050 -0.10520  0.00225  0.10509  0.32770
##
## Coefficients:
##                        Estimate Std. Error t value Pr(>|t|)
## (Intercept)           -2.249764   0.071180 -31.607   <2e-16 ***
## PopulationCaigul       0.005862   0.045563   0.129   0.8979
## PopulationLowerLalaja  0.021406   0.049227   0.435   0.6647
## PopulationTaylor       0.057155   0.045498   1.256   0.2122
## PopulationUpperLalaja -0.031867   0.047352  -0.673   0.5026
## SexMale               -0.097151   0.029674  -3.274   0.0015 **
## log(BrainMass_mg)      0.998782   0.064290  15.536   <2e-16 ***
## ---
## Signif. codes:  0 '***' 0.001 '**' 0.01 '*' 0.05 '.' 0.1 ' ' 1
##
## Residual standard error: 0.1383 on 92 degrees of freedom
## Multiple R-squared:  0.7988, Adjusted R-squared:  0.7856
## F-statistic: 60.86 on 6 and 92 DF,  p-value: < 2.2e-16
##
##
## Response Y2 :
##
## Call:
## lm(formula = Y2 ~ Population + Sex + log(BrainMass_mg), data = ds)
##
## Residuals:
##      Min       1Q   Median       3Q      Max
## -0.34257 -0.07560 -0.00450  0.07776  0.21794
##
## Coefficients:
##                        Estimate Std. Error t value Pr(>|t|)
## (Intercept)           -1.328836   0.055744 -23.838  < 2e-16 ***
## PopulationCaigul       0.015024   0.035682   0.421    0.675
## PopulationLowerLalaja -0.026834   0.038551  -0.696    0.488
## PopulationTaylor      -0.032120   0.035631  -0.901    0.370
## PopulationUpperLalaja  0.009643   0.037083   0.260    0.795
## SexMale                0.184834   0.023239   7.954  4.5e-12 ***
## log(BrainMass_mg)      1.132854   0.050348  22.500  < 2e-16 ***
## ---
## Signif. codes:  0 '***' 0.001 '**' 0.01 '*' 0.05 '.' 0.1 ' ' 1
```

```
##
## Residual standard error: 0.1083 on 92 degrees of freedom
## Multiple R-squared:  0.8599, Adjusted R-squared:  0.8508
## F-statistic: 94.14 on 6 and 92 DF,  p-value: < 2.2e-16
##
##
## Response Y3 :
##
## Call:
## lm(formula = Y3 ~ Population + Sex + log(BrainMass_mg), data = ds)
##
## Residuals:
##      Min       1Q   Median       3Q      Max
## -0.34481 -0.09108  0.01036  0.11412  0.29641
##
## Coefficients:
##                        Estimate Std. Error t value Pr(>|t|)
## (Intercept)           -2.675583   0.076519 -34.966   <2e-16 ***
## PopulationCaigul      -0.028246   0.048981  -0.577    0.566
## PopulationLowerLalaja -0.058214   0.052919  -1.100    0.274
## PopulationTaylor      -0.017748   0.048910  -0.363    0.718
## PopulationUpperLalaja -0.021620   0.050904  -0.425    0.672
## SexMale               -0.001341   0.031900  -0.042    0.967
## log(BrainMass_mg)      1.143957   0.069112  16.552   <2e-16 ***
## ---
## Signif. codes:  0 '***' 0.001 '**' 0.01 '*' 0.05 '.' 0.1 ' ' 1
##
## Residual standard error: 0.1487 on 92 degrees of freedom
## Multiple R-squared:  0.7884, Adjusted R-squared:  0.7745
## F-statistic: 57.11 on 6 and 92 DF,  p-value: < 2.2e-16
##
##
## Response Y4 :
##
## Call:
## lm(formula = Y4 ~ Population + Sex + log(BrainMass_mg), data = ds)
##
## Residuals:
##      Min       1Q   Median       3Q      Max
## -0.47418 -0.11618  0.00906  0.11798  0.41575
##
## Coefficients:
##                        Estimate Std. Error t value Pr(>|t|)
## (Intercept)           -2.638559   0.091287 -28.904   <2e-16 ***
## PopulationCaigul       0.010529   0.058433   0.180    0.857
## PopulationLowerLalaja -0.042250   0.063132  -0.669    0.505
## PopulationTaylor      -0.039678   0.058350  -0.680    0.498
## PopulationUpperLalaja -0.004654   0.060728  -0.077    0.939
## SexMale               -0.065841   0.038057  -1.730    0.087 .
## log(BrainMass_mg)      1.205353   0.082450  14.619   <2e-16 ***
## ---
## Signif. codes:  0 '***' 0.001 '**' 0.01 '*' 0.05 '.' 0.1 ' ' 1
##
## Residual standard error: 0.1774 on 92 degrees of freedom
```

```
## Multiple R-squared:  0.7533, Adjusted R-squared:  0.7372
## F-statistic: 46.81 on 6 and 92 DF,  p-value: < 2.2e-16
##
##
## Response Y5 :
##
## Call:
## lm(formula = Y5 ~ Population + Sex + log(BrainMass_mg), data = ds)
##
## Residuals:
##      Min       1Q   Median       3Q      Max
## -0.41227 -0.12643 -0.04457  0.11612  0.48230
##
## Coefficients:
##                         Estimate Std. Error t value Pr(>|t|)
## (Intercept)           -3.747e+00  1.013e-01 -36.998  < 2e-16 ***
## PopulationCaigul      -9.449e-03  6.484e-02  -0.146    0.884
## PopulationLowerLalaja  3.279e-02  7.005e-02   0.468    0.641
## PopulationTaylor      -5.049e-05  6.474e-02  -0.001    0.999
## PopulationUpperLalaja -2.291e-02  6.738e-02  -0.340    0.735
## SexMale               -2.497e-01  4.223e-02  -5.914 5.63e-08 ***
## log(BrainMass_mg)      1.155e+00  9.148e-02  12.620  < 2e-16 ***
## ---
## Signif. codes:  0 '***' 0.001 '**' 0.01 '*' 0.05 '.' 0.1 ' ' 1
##
## Residual standard error: 0.1968 on 92 degrees of freedom
## Multiple R-squared:  0.7634, Adjusted R-squared:  0.748
## F-statistic: 49.48 on 6 and 92 DF,  p-value: < 2.2e-16
##
##
## Response Y6 :
##
## Call:
## lm(formula = Y6 ~ Population + Sex + log(BrainMass_mg), data = ds)
##
## Residuals:
##      Min       1Q   Median       3Q      Max
## -0.96843 -0.22248  0.00996  0.24819  0.73012
##
## Coefficients:
##                       Estimate Std. Error t value Pr(>|t|)
## (Intercept)           -6.17233    0.17494 -35.282  < 2e-16 ***
## PopulationCaigul      -0.07789    0.11198  -0.696    0.488
## PopulationLowerLalaja -0.14457    0.12099  -1.195    0.235
## PopulationTaylor      -0.02396    0.11182  -0.214    0.831
## PopulationUpperLalaja -0.16477    0.11638  -1.416    0.160
## SexMale               -0.47636    0.07293  -6.532 3.53e-09 ***
## log(BrainMass_mg)      1.13110    0.15801   7.159 1.94e-10 ***
## ---
## Signif. codes:  0 '***' 0.001 '**' 0.01 '*' 0.05 '.' 0.1 ' ' 1
##
## Residual standard error: 0.3399 on 92 degrees of freedom
## Multiple R-squared:  0.6141, Adjusted R-squared:  0.589
## F-statistic:  24.4 on 6 and 92 DF,  p-value: < 2.2e-16
```
